# Activity Coefficients at Infinite Dilution and Physicochemical Properties for Organic Solutes and Water in the Ionic Liquid 1-Ethyl-3-methylimidazolium trifluorotris(perfluoroethyl)phosphate

**DOI:** 10.1007/s10953-014-0274-0

**Published:** 2014-12-13

**Authors:** Michał Wlazło, Andrzej Marciniak, Trevor M. Letcher

**Affiliations:** 1 Department of Physical Chemistry, Faculty of Chemistry, Warsaw University of Technology, Noakowskiego 3, 00-664 Warsaw, Poland; 2 School of Chemistry and Physics, University of KwaZulu-Natal, Howard College Campus, King George V Avenue, Durban, 4041 South Africa

**Keywords:** Activity coefficients at infinite dilution, Ionic liquid, 1-Ethyl-3-methylimidazolium trifluorotris(perfluoroethyl)phosphate, [emim][FAP], Selectivity, LFER

## Abstract

New data of activity coefficients at infinite dilution, *γ*_13_^∞^, for 65 different solutes including alkanes, alkenes, alkynes, cycloalkanes, aromatic hydrocarbons, alcohols, thiophene, ethers, ketones, aldehydes, esters and water in the ionic liquid 1-ethyl-3-methylimidazolium trifluorotris(perfluoroethyl)phosphate, were determined using inverse gas chromatography within the temperature range from 318.15 to 368.15 K. This is a continuation of our study of ionic liquids based on this anion. The results are compared with the other trifluorotris(perfluoroethyl)phosphate ionic liquids. The *γ*_13_^∞^ values were used to calculate thermodynamic functions such as partial molar excess Gibbs energies $\Delta G_{1}^{{{\text{E,}}\infty }}$, enthalpies $\Delta H_{1}^{{{\text{E,}}\infty }}$ and entropies $\Delta S_{1}^{{{\text{E,}}\infty }}$ as well as gas–liquid partition coefficients of the solutes, *K*_L_. These values were used to determine the linear free energy relationship (LFER) system constants as a function of temperature. The selectivities at infinite dilution needed for some extraction problems were calculated and compared with literature data of ionic liquids based on the trifluorotris(perfluoroethyl)phosphate anion and the 1-ethyl-3-methylimidazolium cation. Additionally, the density and viscosity of the investigated ionic liquid at temperatures from 298.15 to 348.15 K were measured.

**Electronic supplementary material:**

The online version of this article (doi:10.1007/s10953-014-0274-0) contains supplementary material, which is available to authorized users.

## Introduction

The negligible vapor pressure of ionic liquids (ILs) makes it possible to consider ILs as environment-friendly compounds that can be used as entrainers in azeotrope breaking and fuel desulphurization, in liquid–liquid extraction processes, and in extractive distillation [1, 2]. The extremely low volatility of ILs not only results in negligible solvent loss to the atmosphere, but also makes it possible to recover ILs by vacuum distillation after extraction, which is an advantage from an economic point of view. The number of azeotropes occurring in industry is large, for instance: water/alcohol systems formed in fermentation processes, alcohol/esters in esterification reactions, alcohol/aliphatic hydrocarbons in petrochemical processes as a result of using alcohols as oxygenated additives for gasolines, aromatic/aliphatic hydrocarbons, aromatic/cyclic hydrocarbons and aliphatic hydrocarbons/sulfur compounds occurring in petroleum, alcohol/ketones and water/THF formed in dehydrocyclization of 1,4-butanediol, and many others. Knowledge of the physical properties of ILs and their interaction with large number of different solvents is necessary to check their potential application in the separation of azeotropes. In addition, it allows one to determine the influence of the structure of ILs properties and consequently the tailoring of ILs for specialized applications. The almost unlimited number of possible combination of anions and cations makes this possible.

Insight into the interactions between ILs and different compounds is given by activity coefficients at infinite dilution *γ*_13_^∞^. From these data, it is possible to calculate the selectivity *S*_12_^∞^ = *γ*_13_^∞^/*γ*_23_^∞^ and capacity *k*_2_^∞^ = 1/*γ*_23_^∞^ at infinite dilution for specific azeotropic mixtures. In this work, we continue a systematic investigation of *γ*_13_^∞^ for trifluorotris(perfluoroethyl)phosphate [FAP]^−^ based ILs, which indicates satisfactory values of *S*_12_^∞^ and *k*_2_^∞^ for many extraction problems [3–8]. In this paper we have chosen 1-ethyl-3-methylimidazolium trifluorotris(perfluoroethyl)phosphate [emim][FAP] and measure the density and the viscosity (over the temperature range of 298.15 to 348.15 K) and *γ*_13_^∞^ (over the temperature range of 318.15 and 368.15 K) for systems involving 65 solutes that include alkanes, alkenes, alkynes, cycloalkanes, aromatic hydrocarbons, alcohols, thiophene, ethers, aldehydes, ketones, esters, 1-nitropropane, acetonitrile and water. The determination of *γ*_13_^∞^ was carried out using inverse gas chromatography, a widely accepted and useful method that is particularly useful for solvents of low volatility such as ILs. Based on these values of *γ*_13_^∞^, gas–liquid partition coefficients, *K*_L_, and basic thermodynamics functions, such as partial molar excess Gibbs energies $\Delta G_{1}^{{{\text{E,}}\infty }}$, enthalpies $\Delta H_{1}^{{{\text{E,}}\infty }}$ and entropies $\Delta S_{1}^{{{\text{E,}}\infty }}$, have been calculated over the relevant temperature range.

The selectivities of [emim][FAP] were calculated for the following azeotropic mixtures: hexane/benzene, cyclohexane/benzene and heptane/thiophene. Because ILs containing the [emim]^+^ cation are among the most studied in the literature, a comparison of selectivities was possible for wide range of ILs based on this cation with different anions [9–20]. Furthermore, a literature review has shown that *γ*_13_^∞^ for [emim][FAP] have previously been determined [21] for alkanes, cycloalkanes, alkanes, alkynes, aromatic hydrocarbon and selected alcohols. Our work expands the investigation to include other classes of compounds as well as allowing us to compare our values of *γ*_13_^∞^ and *S*_12_^∞^ with those obtained by Yan et al. [21]. Additionally, the *γ*_13_^∞^ of [emim][FAP] were compared with [FAP]^−^ based ILs with other imidazolium cations, namely 1-(2-hydroxyethyl)-3-methylimidazolium [C_2_OHmim]^+^ and 1-hexyl-3-methylimidazolium [hmim]^+^ [7, 22]. The selectivities are also compared with those of other [FAP]^−^ ILs [3–5, 22, 23].

## Experimental Method

### Materials

The ionic liquid [emim][FAP] had a purity of >0.990 mass fraction and was supplied by Merck. This ionic liquid was further purified by subjecting the liquid to a very low pressure of about 5 × 10^−3^ Pa at about 363 K for approximately 5 h. This procedure removed any volatile chemicals and water from the ionic liquid. The water content was analyzed by Karl-Fischer titration (method TitroLine KF). The sample of IL was dissolved in methanol and titrated with steps of 2.5 μL. The results obtained showed the water content was less than 200 ppm. The density was measured using an Anton Paar GmbH 4500 vibrating-tube densimeter (Graz, Austria) with an uncertainty ±1 × 10^−5^ g·cm^−3^. Viscosity measurements were performed using an Anton Paar GmbH AMVn Automated Micro Viscometer (Graz, Austria) with an uncertainty ±0.1 %. The solutes, purchased from Aldrich and Fluka, were used without further purification because the IGC technique separated any impurities on the column. The list of materials with purities are presented in the Online Supplementary Material Table 1S. The structure of investigated IL is:





### Apparatus and Experimental Procedure

The experiments were performed using a Perkin Elmer Clarus 500 gas chromatograph equipped with a thermal conductivity detector (TCD). The data were collected and processed using TotalChrom Workstation software. The column preparation and the packing method used in this work have been described previously [24]. Glass columns of length 1 m and 4 mm internal diameter were used. Chromosorb WHP/AW-DMCS 100/120 mesh was used as the solid support and was supplied by Sigma–Aldrich. Coating the solid support material with the ionic liquid was performed by dispersing a certain portion of Chromosorb in a solution of the ionic liquid in methanol followed by evaporation of the solvent using a rotating evaporator. The masses of the stationary phase and of the solid support were weighed with a precision ±0.0001 g. The solvent column packing varied from (45.1–50.0) mass fraction of the ionic liquid, large enough to prevent any residual adsorption of solute on the column packing. The uncertainty in the moles of the IL packed on the support is about ±3 × 10^−7^ mol. Before experiments, each column was conditioned passing carrier gas through it at a flow rate (about 1.5 × 10^−6^ m^3^·s^−1^) at the high temperature (373.15 K) for about 8 h. The second column was used to check the reproducibility of results at a different packing level. On the second column, measurements were performed at two temperatures (338.15 and 358.15) K. Results from these two different columns were reproducible with errors less than 0.5 %.

The pressure drop (*p*_i_–*p*_o_) varied between (20 and 60) kPa, depending on flow rate of carrier gas. The inlet pressure *p*_i_ was measured by a pressure gauge installed on the gas chromatograph with an uncertainty of ±0.1 kPa. The outlet pressure *p*_o_ was measured using an Agilent Precision Gas Flow Meter with an uncertainty of ±0.07 kPa.

The carrier gas was helium. The flow rate of carrier gas was determined using an Agilent Precision Gas Flow Meter, which was placed at the outlet present after the detector, with an uncertainty of ±0.1 × 10^−6^ m^3^·min^−1^. The flow rate was set for a series of runs and was allowed to stabilize for at least 15 min before the retention times were determined. The flow rates were corrected for water vapor pressure. Solute injections ranged from 0.01 to 0.2 μL and were considered to be at infinite dilution on the column.

Experiments were carried out at different temperatures (in steps of 10 K) between 318.15 and 368.15 K. The temperature of the column was maintained constant to within ±0.02 K. At a given temperature, each experiment was repeated 2–3 times to check the reproducibility. Retention times were generally reproducible within (0.001–0.01) min depending on the temperature and the individual solute. At each temperature values of the dead time *t*_G_, identical to the retention time of a non-retainable component, were measured. While our GC was equipped with a TCD detector, air was used as a non-retainable component. The estimated overall error in *γ*_13_^∞^ is less than 3 %, taking into account the possible errors in determining the column loading, the retention times and solute vapor pressure. The IGC technique was tested for the system hexane in hexadecane at *T* = 298.15 K and the results compare very favorably with the literature values [25].

### Theoretical Basis

The equation developed by Everett [26] and Cruickshank et al. [27] was used in this work to calculate the *γ*_13_^∞^ of the solutes in the ionic liquid:

1 $$ \ln \gamma_{13}^{\infty } = \ln \left( {\frac{{n_{3} RT}}{{V_{\text{N}} p_{1}^{*} }}} \right) - \frac{{p_{1}^{*} \left( {B_{11} - V_{1}^{*} } \right)}}{RT} + \frac{{p_{\text{o}} J_{2}^{3} \left( {2B_{12} - V_{1}^{\infty } } \right)}}{RT} $$

The description of the equation and the other equations used in this work to calculate physicochemical properties (*K*_L_, $\Delta G_{1}^{{{\text{E,}}\infty }}$, $\Delta H_{1}^{{{\text{E,}}\infty }}$, $\Delta S_{1}^{{{\text{E,}}\infty }}$) are available in the literature [28]. The densities of the [emim][FAP] needed to calculate *K*_L_ are given in Table 1.

**Table 1:** Density, *ρ*, and viscosity, *η*, as a function of temperature, *T*, for ionic liquid [emim][FAP] at pressure *p* = 0.1 MPa

*T* (K)	*ρ* (g·cm^−3^)	*η (*mPa·s)
298.15	1.70802	57.67
308.15	1.69962	38.76
318.15	1.68453	27.40
328.15	1.67276	20.28
338.15	1.66107	15.58
348.15	1.64940	12.33
358.15	1.63718^a^	
368.15	1.62517^a^	

Standard uncertainties *u* are *u*(*T*) = ±(0.01 and 0.05) for *ρ* and *η*, respectively, *u*(*ρ*) = ±1 × 10^−4^ g·cm^−3^, *u*(*η*) < ±0.1 %, *u*(*p*) = ±1 kPa

^a^Extrapolated values

## Results and Discussion

Table 1 presents the measured densities and viscosities for [emim][FAP], at temperatures from 298.15 to 348.15 K. Additionally, the densities at 358.15 and 368.15 K were extrapolated for further calculation of gas–liquid partition coefficients *K*_L_.

The experimental activity coefficients at infinite dilution for 65 different solutes in [emim][FAP] in temperature range from 318.15 to 368.15 K are presented in Table 2. Compounds with a longer alkyl chain have higher values of *γ*_13_^∞^ in the following group of solutes: alkanes, cycloalkanes, alkenes, alkynes, aromatic hydrocarbons, alcohols, esters, ethers, aldehydes and ketones. The highest *γ*_13_^∞^ are for alkanes, indicating weak interactions between this series of homologous compounds and [emim][FAP]. Lower values of *γ*_13_^∞^ are observed for the corresponding substances having *π*-electrons in their chemical structure. It can be seen that interactions between the IL and a solute increase with increasing number of *π*-electrons in the structure (values of *γ*_13_^∞^ take the order: alkenes > alkynes > aromatic hydrocarbons). It is worth noticing that benzene has *γ*_13_^∞^ values that are below 1, while the remaining investigated aromatic hydrocarbons have *γ*_13_^∞^ values close to unity. Cyclization and isomerization in the structure of a solute results a decrease of the *γ*_13_^∞^ values and an increase in the solubility in IL in comparison to alkanes with the same number of carbon atoms. This is probably due to a packing effect and a smaller molar volume. The same effect is present for primary, secondary and tertiary alcohols, ethers and branches ethers. The presence of oxygen, nitrogen, or sulfur atoms in the structure of polar solute compounds leads to high interactions with [emim][FAP]. The lowest values of *γ*_13_^∞^ (below 1) are for thiophene, pyridine, esters, tetrahydrofuran, 1,4-dioxane, ketones, aldehydes, acetonitrile and 1-nitropropane. Higher *γ*_13_^∞^ values are seen for alcohols, water and ethers. For example the *γ*_13_^∞^ value for di-*n*-butyl ether is even greater than 9.

**Table 2:** The experimental activity coefficients at infinite dilution *γ*_13_^∞^ for the solutes in the ionic liquid [emim][FAP] at different temperatures

Solute	*T* (K)					
	318.15	328.15	338.15	348.15	358.15	368.15
Pentane	11.3	10.8	10.4	10.1	9.70	9.38
Hexane	16.3	15.6	14.9	14.3	13.8	13.3
3-Methylpentane	14.1	13.4	12.9	12.4	12.0	11.6
2,2-Dimethylbutane	12.3	11.8	11.4	11.0	10.7	10.4
Heptane	24.3	22.8	21.5	20.3	19.2	18.3
Octane	36.1	33.4	31.2	29.1	27.3	25.8
2,2,4-Trimethylpentane	21.1	20.0	19.1	18.3	17.5	16.9
Nonane	53.5	48.9	44.8	41.5	38.4	35.7
Decane	79.7	72.1	65.2	59.8	54.9	50.7
Cyclopentane	7.24	6.96	6.70	6.49	6.27	6.08
Cyclohexane	10.8	10.2	9.72	9.27	8.87	8.50
Methylcyclohexane	14.2	13.4	12.7	12.2	11.6	11.1
Cycloheptane	15.5	14.5	13.7	13.0	12.3	11.7
Cyclooctane	21.8	20.2	18.8	17.6	16.5	15.6
Pent-1-ene	5.33	5.30	5.27	5.25	5.22	5.21
Hex-1-ene	7.91	7.76	7.61	7.48	7.36	7.25
Cyclohexene	5.94	5.83	5.72	5.62	5.53	5.45
Hept-1-ene	11.6	11.3	11.1	10.8	10.6	10.4
Oct-1-ene	17.4	16.7	16.1	15.5	15.0	14.6
Dec-1-ene	37.2	35.1	33.3	31.7	30.2	28.9
Pent-1-yne	2.24	2.30	2.36	2.42	2.48	2.53
Hex-1-yne	3.30	3.36	3.41	3.47	3.52	3.57
Hept-1-yne	4.80	4.84	4.88	4.91	4.94	4.98
Oct-1-yne	7.03	7.01	6.99	6.97	6.95	6.93
Benzene	0.782	0.833	0.882	0.935	0.982	1.04
Toluene	1.12	1.20	1.27	1.33	1.41	1.48
Ethylbenzene	1.76	1.84	1.93	2.01	2.10	2.18
*o*-Xylene	1.54	1.62	1.70	1.79	1.87	1.96
*m*-Xylene	1.65	1.75	1.84	1.94	2.03	2.12
*p*-Xylene	1.72	1.81	1.90	1.99	2.08	2.17
Styrene	1.09	1.16	1.23	1.30	1.37	1.44
*α*-Methylstyrene	1.72	1.84	1.96	2.08	2.20	2.32
Thiophene	0.847	0.891	0.935	0.976	1.02	1.06
Pyridine	0.417	0.444	0.472	0.498	0.526	0.553
Methanol	2.24	2.08	1.94	1.82	1.71	1.61
Ethanol	2.64	2.43	2.24	2.09	1.94	1.82
Propan-1-ol	3.40	3.11	2.85	2.63	2.44	2.27
Propan-2-ol	2.65	2.43	2.25	2.09	1.95	1.83
Butan-1-ol	4.49	4.05	3.68	3.36	3.09	2.85
Butan-2-ol	3.17	2.91	2.69	2.49	2.32	2.18
2-Methyl-propan-1-ol	4.20	3.78	3.39	3.10	2.83	2.60
*tert*-Butanol	2.24	2.11	1.99	1.89	1.80	1.72
Pentan-1-ol	5.39	4.90	4.48	4.12	3.81	3.54
Water	6.73	6.04	5.46	4.95	4.53	4.16
Methyl acetate	0.371	0.401	0.432	0.462	0.494	0.526
Methyl propanoate	0.486	0.524	0.564	0.605	0.645	0.687
Methyl butanoate	0.689	0.737	0.786	0.836	0.886	0.937
Ethyl acetate	0.486	0.523	0.561	0.599	0.639	0.678
Vinyl acetate	0.671	0.708	0.742	0.778	0.813	0.847
Tetrahydrofuran	0.472	0.507	0.541	0.576	0.613	0.649
1,4-Dioxane	0.354	0.391	0.427	0.465	0.503	0.543
*tert*-Butyl methyl ether	1.38	1.49	1.59	1.69	1.80	1.91
*tert*-Butyl ethyl ether	3.13	3.26	3.39	3.52	3.65	3.78
*tert*-Amyl methyl ether	2.12	2.25	2.37	2.50	2.62	2.74
Diethyl ether	1.48	1.56	1.64	1.73	1.81	1.89
Di-*n*-propyl ether	4.15	4.24	4.33	4.41	4.49	4.57
Di-*iso*-propyl ether	3.25	3.42	3.58	3.74	3.89	4.06
Di-*n*-butyl ether	9.35	9.29	9.23	9.18	9.13	9.08
Acetone	0.237	0.257	0.276	0.297	0.318	0.338
Pentan-2-one	0.420	0.452	0.485	0.518	0.552	0.586
Pentan-3-one	0.430	0.466	0.501	0.537	0.573	0.610
Propanal	0.367	0.392	0.417	0.443	0.469	0.494
Butanal	0.496	0.527	0.56	0.591	0.625	0.656
Acetonitrile	0.308	0.325	0.34	0.357	0.373	0.389
1-Nitropropane	0.583	0.604	0.624	0.643	0.663	0.682

Standard uncertainties *u* are *u*(*γ*_13_^∞^) < ±3 %, *u*(*T*) = ±0.02 K

Table 3 shows the gas–liquid partition coefficients *K*_L_ of solutes calculated from measured *γ*_13_^∞^ and densities for [emim][FAP]. These values decrease with increasing temperature in all cases and increase with increasing alkyl chain length for all of the investigated groups of solutes. The highest value was observed for 1-nitropropane. Other determined thermodynamic functions, namely partial molar excess Gibbs energies $\Delta G_{1}^{{{\text{E,}}\infty }}$, enthalpies $\Delta H_{1}^{{{\text{E,}}\infty }}$ and entropies $\Delta S_{1}^{{{\text{E,}}\infty }}$ at infinite dilution are presented at temperature 328.15 K in Table 4. Alkynes, aromatic hydrocarbons, thiophene, pyridine, esters, ethers, ketones, aldehydes, acetonitrile and 1-nitropropane reveal stronger interactions between the IL and the solute pairs than between the solutes themselves, which show negative values of $\Delta H_{1}^{{{\text{E,}}\infty }}$. The exceptions are oct-1-yne and di-*n*-butyl ether with positive $\Delta H_{1}^{{{\text{E,}}\infty }}$ due to their more aliphatic character. Positive values of $\Delta S_{1}^{{{\text{E,}}\infty }}$ are seen for alcohols and water, which indicates a breaking of hydrogen bonds during the dissolution process.

**Table 3:** The experimental gas–liquid partition coefficients *K*_L_ for solutes in the ionic liquid [emim][FAP] at different temperatures

Solute	*T* (K)					
	318.15	328.15	338.15	348.15	358.15	368.15
Pentane	5.51	4.39	3.56	2.93	2.46	2.09
Hexane	11.2	8.46	6.56	5.19	4.18	3.42
3-Methylpentane	10.7	8.20	6.39	5.09	4.13	3.40
2,2-Dimethylbutane	7.90	6.18	4.94	4.01	3.32	2.78
Heptane	21.9	15.9	11.9	9.09	7.10	5.63
Octane	42.3	29.5	21.2	15.6	11.8	9.04
2,2,4-Trimethylpentane	24.2	17.6	13.1	10.1	7.83	6.22
Nonane	80.9	54.2	37.5	26.6	19.5	14.5
Decane	154	98.8	66.0	45.1	31.8	23.0
Cyclopentane	13.0	10.1	7.98	6.39	5.23	4.34
Cyclohexane	25.3	18.9	14.5	11.3	8.99	7.28
Methylcyclohexane	37.9	27.6	20.6	15.7	12.2	9.70
Cycloheptane	68.0	48.4	35.3	26.3	20.1	15.6
Cyclooctane	164	112	78.2	56.1	41.4	31.0
Pent-1-ene	9.70	7.50	5.92	4.76	3.89	3.23
Hex-1-ene	19.6	14.4	10.9	8.43	6.64	5.32
Cyclohexene	49.4	35.6	26.3	19.9	15.3	12.0
Hept-1-ene	38.0	26.9	19.5	14.6	11.1	8.62
Oct-1-ene	73.1	49.7	34.7	25.0	18.4	13.9
Dec-1-ene	261	164	107	71.6	49.4	34.9
Pent-1-yne	31.5	23.0	17.2	13.1	10.2	8.10
Hex-1-yne	61.6	43.1	31.0	22.9	17.2	13.3
Hept-1-yne	120	80.5	55.8	39.7	28.9	21.5
Oct-1-yne	230	148	98.8	67.8	47.8	34.5
Benzene	350	23	158	110	79.1	57.8
Toluene	730	460	300	202	139	98.4
Ethylbenzene	1,242	757	479	312	210	144
*o*-Xylene	1,975	1,180	731	467	309	209
*m*-Xylene	1,491	898	561	362	240	164
*p*-Xylene	1,376	834	525	340	227	156
Styrene	2,961	1,730	1,057	668	436	292
*α*-Methylstyrene	4,123	2,351	1,394	855	543	354
Thiophene	377	249	170	119	85.8	63.2
Pyridine	2,539	1,561	990	651	439	305
Methanol	82.1	58.9	43.4	32.7	25.1	19.6
Ethanol	133	92.3	65.7	47.9	35.9	27.4
Propan-1-ol	257	170	116	82.4	59.7	44.5
Propan-2-ol	168	113	78.2	56.1	41.4	31.3
Butan-1-ol	529	336	221	150	105	76.3
Butan-2-ol	313	203	136	95.0	68.2	50.4
2-Methyl-propan-1-ol	368	235	158	109	77.6	57.0
*tert*-Butanol	200	131	88.9	62.6	45.5	34.1
1-Pentanol	1,095	661	417	273	184	128
Water	124	86.6	61.8	45.3	33.9	25.8
Methyl acetate	342	227	155	109	78.4	57.9
Methyl propanoate	596	380	250	170	119	84.9
Methyl butanoate	1,012	625	399	263	179	125
Ethyl acetate	550	352	233	159	111	80.2
Vinyl acetate	336	222	151	106	76.1	56.1
Tetrahydrofuran	356	237	163	115	82.9	61.2
1,4-Dioxane	1,766	1,080	685	448	301	208
*tert*-Butyl methyl ether	84.1	57.5	40.4	29.2	21.6	16.4
*tert*-Butyl ethyl ether	67.9	46.3	32.5	23.5	17.4	13.1
*tert*-Amyl methyl ether	160	106	71.9	50.3	36.1	26.5
Diethyl ether	39.6	28.4	20.9	15.7	12.1	9.43
Di-*n*-propyl ether	96.0	64.7	45.0	32.1	23.5	17.6
Di-*iso*-propyl ether	57.0	38.8	27.2	19.6	14.5	10.9
Di-*n*-butyl ether	331	206	133	88.7	60.8	42.9
Acetone	517	345	238	168	121	89.7
Pentan-2-one	1,554	966	621	411	280	196
Pentan-3-one	1,482	922	594	393	268	187
Ppropanal	251	173	123	89.0	65.9	49.9
Butanal	472	313	214	150	108	79.7
Acetonitrile	968	648	448	316	228	168
1-Nitropropane	3,497	2,143	1,361	893	602	417

**Table 4:** Limiting partial molar excess Gibbs energies, $\Delta G_{1}^{{{\text{E}},\infty }}$, enthalpies $\Delta H_{1}^{{{\text{E}},\infty }}$, and entropies $T_{\text{ref}} \Delta S_{1}^{{{\text{E}},\infty }}$ for the solutes in the ionic liquid [emim][FAP] at the reference temperature *T*_ref_ = 328.15 K

Solute	$\Delta G_{1}^{{{\text{E}},\infty }}$ (kJ·mol^−1^)	$\Delta H_{1}^{{{\text{E}},\infty }}$ (kJ·mol^−1^)	$T_{\text{ref}} \Delta S_{1}^{{{\text{E}},\infty }}$ (kJ·mol^−1^)
Pentane	6.49	3.7	−2.83
Hexane	7.50	4.1	−3.44
3-Methylpentane	7.08	3.7	−3.37
2,2-Dimethylbutane	6.73	3.2	−3.53
Heptane	8.53	5.6	−2.94
Octane	9.57	6.6	−3.01
2,2,4-Trimethylpentane	8.17	4.3	−3.82
Nonane	10.61	7.8	−2.77
Decane	11.67	8.8	−2.85
Cyclopentane	5.29	3.4	−1.92
Cyclohexane	6.34	4.6	−1.73
Methylcyclohexane	7.08	4.7	−2.37
Cycloheptane	7.30	5.5	−1.83
Cyclooctane	8.20	6.5	−1.65
Pent-1-ene	4.55	0.5	−4.10
Hex-1-ene	5.59	1.7	−3.88
Cyclohexene	4.81	1.7	−3.12
Hept-1ene	6.62	2.1	−4.47
Oct-1-ene	7.68	3.5	−4.22
Dec-1-ene	9.71	4.9	−4.78
Pent-1-yne	2.27	−2.4	−4.66
Hex-1-yne	3.31	−1.5	−4.85
Hept-1-ene	4.30	−0.7	−4.99
Oct-1-yne	5.31	0.3	−5.02
Benzene	−0.50	−5.4	−4.94
Toluene	0.50	−5.4	−5.85
Ethylbenzene	1.66	−4.2	−5.86
*o*-Xylene	1.32	−4.7	−6.00
*m*-Xylene	1.53	−4.8	−6.37
*p*-Xylene	1.62	−4.5	−6.10
Styrene	0.40	−5.5	−5.86
*α*-Methylstyrene	1.66	−5.8	−7.49
Thiophene	−0.31	−4.4	−4.06
Pyridine	−2.22	−5.5	−3.27
Methanol	2.00	6.4	4.43
Ethanol	2.42	7.3	4.83
Propan-1-ol	3.10	7.8	4.75
Propan-2-ol	2.42	7.2	4.79
Butan-1-ol	3.82	8.9	5.05
Butan-2-ol	2.91	7.4	4.44
2-Methyl-propan-1-ol	3.63	9.3	5.72
*tert*-Butanol	2.04	5.2	3.12
1-Pentanol	4.34	8.2	3.87
Water	4.91	9.4	4.49
Methyl acetate	−2.49	−6.8	−4.29
Methyl propanoate	−1.76	−6.7	−4.97
Methyl butanoate	−0.83	−6.0	−5.14
Ethyl acetate	−1.77	−6.5	−4.73
Vinyl acetate	−0.94	−4.5	−3.60
Tetrahydrofuran	−1.85	−6.2	−4.36
1,4-Dioxane	−2.56	−8.3	−5.74
*tert*-Butyl methyl ether	1.09	−6.3	−7.37
*tert*-Butyl ethyl ether	3.22	−3.7	−6.92
*tert*-Amyl methyl ether	2.21	−5.0	−7.21
Diethyl ether	1.21	−4.8	−6.02
di-*n*-Propyl ether	3.94	−1.9	−5.80
di-*iso*-Propyl ether	3.35	−4.3	−7.67
di-*n*-Butyl ether	6.08	0.6	−5.51
Acetone	−3.71	−6.9	−3.19
Pentan-2-one	−2.17	−6.5	−4.33
Pentan-3-one	−2.08	−6.8	−4.71
Propanal	−2.56	−5.8	−3.23
Butanal	−1.75	−5.5	−3.72
Acetonitrile	−3.07	−4.6	−1.50
1-Nitropropane	−1.38	−3.0	−1.65

**Fig. 1:**
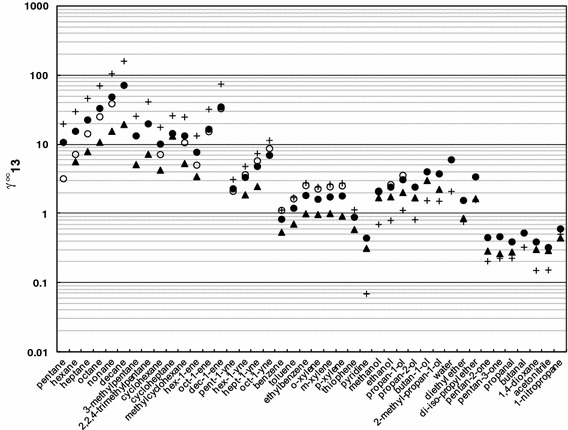
Comparison of *γ*_13_^∞^ at *T* = 328.15 K for selected solutes in ionic liquids: (*filled circle*) [emim][FAP], (*empty circle*) [emim][FAP] [21], (*filled triangles*) [hmim][FAP] [22], (+) [C_2_OHmim][FAP] [6]

Figure 1 shows a comparison of *γ*_13_^∞^ values at 328.15 K obtained in this work for selected solutes and [emim][FAP], together with other ILs based on the [FAP]^−^ anion and imidazolium cation: the IL [hmim][FAP], which differs from the IL investigated in this work with a hexyl group replacing the ethyl group in the cation structure, and [C_2_OHmim][FAP], which has an hydroxyl group in the ethyl side-chain of the cation [7, 22]. The full names and the structures of these ILs as well as other ILs used in the comparative studies of selectivities are included in the Online Supplementary Material Table 2S. Moreover, previously published data for [emim][FAP] are also presented in Fig. 1 [21]. The IL investigated in this work, [emim][FAP], reveals higher values of *γ*_13_^∞^ than the IL [hmim][FAP], for all compared solutes. The difference is greater in the case of alkanes, alkenes, alkynes, cycloalkanes and aromatic hydrocarbons. This is no doubt caused by the stronger interactions between IL and the more non-polar alkyl chain and non-polar compounds. The IL [C_2_OHmim][FAP], with its polar hydroxyl group, has the highest *γ*_13_^∞^ values for non-polar aliphatic and aromatic hydrocarbons and the lowest ones for polar compounds.

**Fig. 2:**
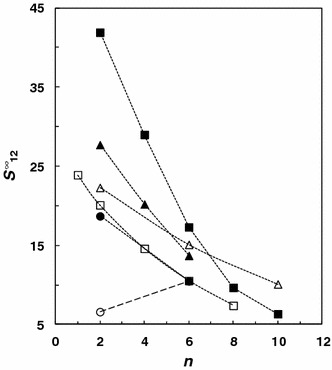
Comparison of selectivity, *S*_12_^∞^, at *T* = 328.15 K for hexane/benzene separation problem of different 1-alkyl-3-methylimidazolium ionic liquids: (*filled circle*) [FAP]^−^ this work and [22], (*empty circle*) [FAP]^−^ [21, 22], (*empty square*) [NTf_2_]^−^ [10, 29–31]; (*empty triangle*) [TCB]^−^ [11, 32, 33], (*filled triangle*) [CF_3_SO_3_]^−^ [14, 34, 35], (*filled square*) [BF_4_]^−^ [18, 36–39], where alkyl = *n*-C_*n*_H_2*n*+1_. The* lines* are drawn to guide the eye

**Fig. 3:**
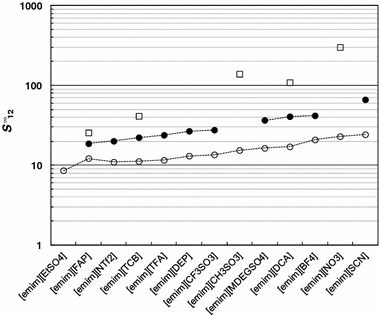
Selectivity, *S*_12_^∞^, at *T* = 328.15 K for ionic liquids based on [emim]^+^ cation for separation: (*filled circle*) hexane/benzene [10–15, 17, 18, 20]; (*empty circle*) cyclohexane/benzene [9–20], (*empty square*) heptane/thiophene [11, 15, 17, 19]. The* lines* are drawn to guide the eye

**Fig. 4:**
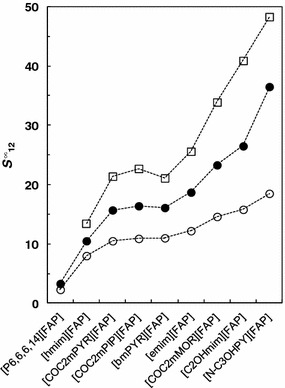
Selectivity, *S*_12_^∞^, at *T* = 328.15 K for ionic liquids based on [FAP]^−^ anion for separation: (*filled circle*) hexane/benzene [3–8, 22, 23], (*empty circle*) cyclohexane/benzene [3–8, 22, 23], (*empty square*) heptane/thiophene [3–8, 22]. The *lines* are drawn to guide the eye

In comparison with work of Yan et al. [21], our results are higher than those obtained previously for alkanes. This difference decreases with increasing alkyl chain length. The *γ*_13_^∞^ values obtained in this work are also higher for cyclohexane and hex-1-ene but comparable for oct-1-ene and dec-1-ene. In the case of alkynes our results show slightly higher values for pent-1-yne and slightly lower values for hex-1-yne, hep-1-yne and oct-1-yne. Previous measurements reveal higher *γ*_13_^∞^ values for aromatic hydrocarbons and slightly higher values for ethanol and propan-1-ol. These results have a direct impact on the selectivity *S*_12_^∞^ and the capacity *k*_2_^∞^ at infinite dilution for the extraction and separation of aliphatic/aromatic hydrocarbons. The selectivity for [emim][FAP] for the hexane/benzene system as determined in this work is equal to 18.7 at 328.15 K and is higher than that for [hmim][FAP] (*γ*_13_^∞^ = 10.5), while the selectivity calculated from the data of Yan et al. for the same system is lower (*γ*_13_^∞^ = 6.61). The typical behavior for these systems involving imidazolium ILs is that the selectivity, related to the hexane/benzene system, decreases with increasing of number of carbons in the alkyl side-chain. This can be seen in Fig. 2 [10, 11, 14, 18, 21, 22, 29–39]. The reason for this is that the longer the non-polar alkyl chain, the stronger will be the van der Waals interactions with hexane and benzene, thus reducing the difference between IL-hexane and IL-benzene selectivities. Moreover, the longer the alkyl chains are, the more they inhibit the π–π interactions between the aromatic ring of benzene and the imidazole center as a result of steric hinderance. Figure 3 presents a comparison of the selectivity of ILs based on the [emim]^+^ cation for three extraction problems: hexane/benzene, cyclohexane/benzene and heptane/thiophene at 328.15 K [9–20]. Unfortunately, the selectivity for [emim][FAP] is the lowest among the reported ILs for hexane/benzene and heptane/thiophene systems. In the case of cyclohexane/benzene, the situation is significantly better and the selectivity is higher than for [EtSO_4_]^−^, [NTf_2_]^−^, [TCB]^−^ and [TFA]^−^. Figure 4 shows a comparison of the selectivity of [FAP]^−^ based ILs at 328.15 K obtained from the literature for the three above mentioned extraction problems. It can be seen that only three ILs with polar functional groups have higher *S*_12_^∞^ values for these systems than the one investigated in this work, these ILs are [COC_2_mMOR][FAP], [C_2_OHmim][FAP] and [*N*-C_3_OHPY][FAP]. Comparing the *S*_12_^∞^ values for [emim][FAP] and [C_2_OHmim][FAP], it can be concluded that presence of a hydroxyl group in the side chain significantly increases the selectivities due to its stronger interactions with π-electrons of the aromatic ring of benzene or with the sulfur atom in thiophene. However, [emim][FAP] has higher selectivities than do [COC_2_mPYR][FAP] or [COC_2_mPIP][FAP]. This means that the imidazolium cation interacts much more strongly than with benzene and thiophene than do ILs based on pyrrolidinium and piperidinium despite the presence of the methoxy groups in their structures.

The Abraham solvation parameter model [40]:

2 $$ \text{log}_{10} K_{\text{L}} = c + eE + sS + aA + bB + lL $$

gives an estimation of *K*_L_ for additional solutes in [COC_2_mPIP][NTf_2_]. The independent variables in Eq. 2 are solute descriptors described previously [40–42]: *E* is the solute excess molar refraction, *S* is the solute dipolarity/polarizability, *A* and *B* are the overall or summation of solute hydrogen bond acidity and basicity, and *L* is the logarithm of the gas–hexadecane partition coefficient at *T* = 298 K. Solute descriptors are available for wide range of compounds. The six regression coefficients (*c*, *e*, *s*, *a*, *b* and *l*) relate to the properties of the solvent phase and are determined by regression analysis from experimental *K*_L_ values. The *c* coefficient is the model constant taking into account opposing contributions of different effects: *e*—interactions with lone pair electrons, *s* – dipole-type interactions, *a* and *b*—the hydrogen-bond basicity and acidity of the stationary phase respectively, and *l*—cavity formation and dispersion interactions. The linear solvation energy relationship (LFER) system constants as a function of temperature for ionic liquid investigated in this work are presented in the Table 5.

**Table 5:** LFER system constants as a function of temperature for the ionic liquid [emim][FAP]

*T*/K	System constants^a^						Statistics			
	*l*	*b*	*a*	*s*	*e*	*c*	*r* ^2^	SD	*F*	*df*
318.15	0.557 (0.018)	0.62 (0.07)	1.20 (0.08)	2.41 (0.07)	–0.03 (0.07)	–0.40 (0.06)	0.988	0.08	939	59
328.15	0.521 (0.016)	0.56 (0.07)	1.13 (0.08)	2.33 (0.07)	–0.02 (0.07)	–0.43 (0.05)	0.988	0.08	997	59
338.15	0.488 (0.015)	0.52 (0.06)	1.06 (0.07)	2.25 (0.06)	–0.02 (0.06)	–0.45 (0.05)	0.989	0.07	1,055	59
348.15	0.457 (0.014)	0.48 (0.058)	1.00 (0.07)	2.17 (0.06)	–0.01 (0.06)	–0.47 (0.05)	0.989	0.07	1,106	59
358.15	0.429 (0.013)	0.44 (0.05)	0.95 (0.06)	2.10 (0.05)	–0.01 (0.05)	–0.49 (0.04)	0.990	0.06	1,151	59
368.15	0.402 (0.013)	0.40 (0.05)	0.90 (0.06)	2.03 (0.05)	0.00 (0.05)	–0.51 (0.04)	0.990	0.06	1,184	59

*r* ^2^ the coefficient of determination, *SD* the standard deviation, *F* the F statistic, *df* the degrees of freedom

^a^Values in parentheses are standard uncertainties of the parameters

## Conclusions

Activity coefficients at infinite dilution and the gas–liquid partition coefficients for 65 solutes in the ionic liquid 1-ethyl-3-methylimidazolium trifluorotris(perfluoroethyl)phosphate were measured by inverse gas chromatography at temperatures ranging from 318.15 to 368.15 K.

Our results show a higher selectivity of [emim][FAP] for the hexane/benzene system in comparison to data obtained by Yan et al. [21]. The selectivity values calculated in this work from *γ*_13_^∞^ are higher than for [hmim][FAP], which is to be expected for ILs with other anions, while the selectivities calculated using *γ*_13_^∞^ from the Yan et al. work are lower than for [hmim][FAP].

The *γ*_13_^∞^ values for [emim][FAP] reveals the lowest selectivities for ILs based on the [emim]^+^ cation for hexane/benzene and heptane/thiophene extraction problems. The IL investigated in this work has higher values of selectivities for separating cyclohexane/benzene than the ILs [emim][EtSO_4_], [emim][NTf_2_], [emim][TCB] and [emim][TFA].

Among ILs based on the [FAP] anion, only [COC_2_mMOR][FAP], [C_2_OHmim][FAP] and [*N*-C_3_OHPY][FAP] have higher selectivities for the extraction systems studied in this work, compared with the IL [emim][FAP] investigated here. This IL has even higher selectivities than [COC_2_mPIP][FAP] and [COC_2_mPYR][FAP].

## Electronic Supplementary Material

Below is the link to the electronic supplementary material.

Supplementary material 1 (DOC 377 kb)
