# Supplementary material for: Activity Coefficients at Infinite Dilution and Physicochemical Properties for Organic Solutes and Water in the Ionic Liquid 1-Ethyl-3-methylimidazolium trifluorotris(perfluoroethyl)phosphate
Source: J Solution Chem. 2014 Dec 13;44(3):413–30. doi: 10.1007/s10953-014-0274-0 (PMC4412692; doi:10.1007/s10953-014-0274-0)
Supplement: Supplementary file 1 — Supplementary material 1 (DOC 377 kb) [file 10953_2014_274_MOESM1_ESM.doc]

**Journal of Solution Chemistry**

**Electronic supplementary material for:**

**Activity Coefficients at Infinite Dilution and Physicochemical Properties for Organic Solutes and Water in the Ionic Liquid 1-Ethyl-3-methylimidazolium Trifluorotris(perfluoroethyl)phosphate**

**Michał Wlazło · Andrzej Marciniak · Trevor M. Letche**r

**Table 1S** The sources and mass fraction purities of materials

| Chemical name*^a^* | Source | Initial mass fraction purity | Purification method | Final mass fraction purity | Analysis method |
| --- | --- | --- | --- | --- | --- |
| pentane | Aldrich | ≥ 0.99 | – | – | – |
| hexane | Fluka | ≥ 0.99 | – | – | – |
| 3-methylpentane | Aldrich | ≥ 0.99 | – | – | – |
| 2,2-dimethylbutane | Aldrich | ≥ 0.99 | – | – | – |
| heptane | Aldrich | ≥ 0.995 | – | – | – |
| octane | Aldrich | ≥ 0.99 | – | – | – |
| 2,2,4-trimethylpentane | Aldrich | ≥ 0.99 | – | – | – |
| nonane | Aldrich | ≥ 0.99 | – | – | – |
| decane | Aldrich | ≥ 0.99 | – | – | – |
| cyclopentane | Aldrich | ≥ 0.99 | – | – | – |
| cyclohexane | Aldrich | ≥ 0.99 | – | – | – |
| methylcyclohexane | Aldrich | ≥ 0.99 | – | – | – |
| cycloheptane | Aldrich | ≥ 0.98 | – | – | – |
| cyclooctane | Fluka | ≥ 0.99 | – | – | – |
| pent-1-ene | Aldrich | ≥ 0.985 | – | – | – |
| hex-1-ene | Aldrich | ≥ 0.99 | – | – | – |
| cyclohexene | Aldrich | ≥ 0.99 | – | – | – |
| hept-1-ene | Aldrich | ≥ 0.99 | – | – | – |
| oct-1-ene | Aldrich | ≥ 0.98 | – | – | – |
| dec-1-ene | Aldrich | ≥ 0.97 | – | – | – |
| pent-1-yne | Aldrich | ≥ 0.99 | – | – | – |
| hex-1-yne | Aldrich | ≥ 0.97 | – | – | – |
| hept-1-yne | Aldrich | ≥ 0.98 | – | – | – |
| oct-1-yne | Aldrich | ≥ 0.97 | – | – | – |
| benzene | Aldrich | ≥ 0.998 | – | – | – |
| toluene | Aldrich | ≥ 0.998 | – | – | – |
| ethylbenzene | Aldrich | ≥ 0.998 | – | – | – |
| *o*-xylene | Aldrich | ≥ 0.99 | – | – | – |
| *m*-xylene | Aldrich | ≥ 0.99 | – | – | – |
| *p*-xylene | Aldrich | ≥ 0.99 | – | – | – |
| styrene | Aldrich | ≥ 0.999 | – | – | – |
| prop-1-en-2-ylbenzene  (*α*-methylstyrene) | Aldrich | ≥ 0.99 | – | – | – |
| thiophene | Aldrich | ≥ 0.99 | – | – | – |
| pyridine | Aldrich | ≥ 0.998 | – | – | – |
| methanol | Aldrich | ≥ 0.999 | – | – | – |
| ethanol | Aldrich | ≥ 0.998 | – | – | – |
| propan-1-ol | Aldrich | ≥ 0.999 | – | – | – |
| propan-2-ol | Fluka | ≥ 0.999 | – | – | – |
| butan-1-ol | Aldrich | ≥ 0.998 | – | – | – |
| butan-2-ol | Aldrich | ≥ 0.995 | – | – | – |
| 2-methyl-propan-1-ol | Aldrich | ≥ 0.995 | – | – | – |
| 2-methyl-propan-2-ol  (*tert*-butanol) | Aldrich | ≥ 0.997 | – | – | – |
| 1-pentanol | Aldrich | ≥ 0.998 | – | – | – |
| water | own source | – | distillation, filtration | ≥ 0.999 | density |
| methyl acetate | Aldrich | ≥ 0.998 | – | – | – |
| methyl propanoate | Aldrich | ≥ 0.99 | – | – | – |
| methyl butanoate | Aldrich | ≥ 0.99 | – | – | – |
| ethyl acetate | Aldrich | ≥ 0.998 | – | – | – |
| oxolane  (tetrahydrofuran, THF) | Aldrich | ≥ 0.999 | – | – | – |
| 1,4-dioxane | Aldrich | ≥ 0.998 | – | – | – |
| 2-methoxy-2-methylpropane  (*tert*-butyl methyl ether, MTBE) | Aldrich | ≥ 0.998 | – | – | – |
| 2-ethoxy-2-methylpropane  (*tert*-butyl ethyl ether, ETBE) | Aldrich | ≥ 0.99 | – | – | – |
| 2-methoxy-2-methylbutane  (*tert*-amyl methyl ether, TAME) | Aldrich | ≥ 0.97 | – | – | – |
| ethoxyethane  (diethyl ether) | Aldrich | ≥ 0.999 | – | – | – |
| 1-propoxypropane  (di-*n*-propyl ether) | Aldrich | ≥ 0.99 | – | – | – |
| 2-propan-2-yloxypropane  (di-*iso*-propyl ether) | Fluka | ≥ 0.99 | – | – | – |
| 1-butoxybutane  (di-*n*-butyl ether) | Aldrich | ≥ 0.993 | – | – | – |
| propan-2-one  (acetone) | Aldrich | ≥ 0.999 | – | – | – |
| pentan-2-one | Aldrich | ≥ 0.99 | – | – | – |
| pentan-3-one | Aldrich | ≥ 0.99 | – | – | – |
| propanal | Aldrich | ≥ 0.97 | – | – | – |
| butanal | Fluka | ≥ 0.99 | – | – | – |
| acetonitrile | Fluka | ≥ 0.999 | – | – | – |
| 1-nitropropane | Aldrich | ≥ 0.985 | – | – | – |
| trisfluorotris(perfluoroethyl)phosphate; 1-ethyl-3-methylimidazol-3-ium  (1-ethyl-3-methylimidazolium trisfluorotris(perfluoroethyl)phosphate) | Merck | ≥ 0.995 | vacuum heating | – | – |

*^a^* Names in parentheses are common names used in text

**Table 2S** List of the ILs used in comparison of $\gamma_{13}^{\infty}$ and selectivities with abbreviations, names and cation or anion structures

| Abbreviation | Name | Cation or anion structure | Ref. |
| --- | --- | --- | --- |
| [P_6,6,6,14_][FAP] | trihexyl-tetradecyl-phosphonium trifluorotris(perfluoroethyl)phosphate |  | [23] |
| [hmim][FAP] | 1-hexyl-3-methyl-imidazolium trifluorotris(perfluoroethyl)phosphate |  | [22] |
| [COC_2_mPYR][FAP] | 1-(2-methoxyethyl)-1-methylpyrrolidinium trifluorotris(perfluoroethyl)phosphate |  | [3] |
| [COC_2_mPIP][FAP] | 1-(2-methoxyethyl)-1-methylpiperidinium trifluorotris(perfluoroethyl)phosphate |  | [4] |
| [bmPYR][FAP] | 1-butyl-1-methyl-pyrrolidinium trifluorotris(perfluoroethyl)phosphate |  | [5] |
| [COC_2_mMOR][FAP] | 4-(2-methoxyethyl)-4-methylmorpholinium trifluorotris(perfluoroethyl)phosphate |  | [6] |
| [C_2_OHmim][FAP] | 1-(2-hydroxyethyl)-3-methylimidazolium trifluorotris(perfluoroethyl)phosphate |  | [7] |
| [*N*-C_3_OHPY][FAP] | 1-(3-hydroxypropyl)pyridinium trifluorotris(perfluoroethyl)phosphate |  | [8] |
| [emim][EtSO4] | 1-ethyl-3-methylimidazolium ethyl sulfate |  | [9] |
| [emim][NTf_2_] | 1-ethyl-3-methylimidazolium bis(trifluoromethylsulfonyl)amide |  | [10] |
| [emim][TCB] | 1-ethyl-3-methylimidazolium tetracyanoborate |  | [11] |
| [emim][TFA] | 1-ethyl-3-methylimidazolium trifluororacetate |  | [12] |
| [emim][DEP] | 1-ethyl-3-methylimidazolium diethylphosphate |  | [13] |
| [emim][CF_3_SO_3_] | 1-ethyl-3-methylimidazolium trifluoromethanesulfonate |  | [14] |
| [emim][CH_3_SO_3_] | 1-ethyl-3-methylimidazolium methanesulfonate |  | [15] |
| [emim][MDEGSO_4_] | 1-ethyl-3-methylimidazolium 2-(2-methoxyethoxy)ethylsulfate |  | [16] |
| [emim][DCA] | 1-ethyl-3-methylimidazolium dicyanamide |  | [17] |
| [emim][BF_4_] | 1-ethyl-3-methylimidazolium tetrafluoroborate |  | [18] |
| [emim][NO_3_] | 1-ethyl-3-methylimidazolium nitrate |  | [19] |
| [emim][SCN] | 1-ethyl-3-methylimidazolium thiocyanate |  | [20] |
| [mmim][NTf_2_] | 1,3-dimethylimidazolium bis(trifluoromethylsulfonyl)amide |  | [10] |
| [bmim][NTf_2_] | 1-butyl-3-methylimidazolium bis(trifluoromethylsulfonyl)amide |  | [29] |
| [hmim][NTf_2_] | 1-hexyl-3-methylimidazolium bis(trifluoromethylsulfonyl)amide | presented above | [30] |
| [omim][NTf_2_] | 1-octyl-3-methylimidazolium bis(trifluoromethylsulfonyl)amide |  | [31] |
| [hmim][TCB] | 1-hexyl-3-methylimidazolium tetracyanoborate | presented above | [32] |
| [dmim][TCB] | 1-decyl-3-methylimidazolium tetracyanoborate |  | [33] |
| [bmim][CF_3_SO_3_] | 1-butyl-3-methylimidazolium trifluoromethanesulfonate | presented above | [34] |
| [hmim][CF_3_SO_3_] | 1- hexyl-3-methylimidazolium trifluoromethanesulfonate | presented above | [35] |
| [bmim][BF_4_] | 1-butyl-3-methylimidazolium tetrafluoroborate | presented above | [36] |
| [hmim][BF_4_] | 1-hexyl-3-methylimidazolium tetrafluoroborate | presented above | [37] |
| [omim][BF_4_] | 1-octyl-3-methylimidazolium tetrafluoroborate | presented above | [38] |
| [dmim][BF_4_] | 1-decyl-3-methylimidazolium tetrafluoroborate | presented above | [39] |

**Table 3S** Solute descriptors for Eq, 2 [40–42]

| Solute | *E* | *S* | *A* | *B* | *L* |
| --- | --- | --- | --- | --- | --- |
| pentane | 0.000 | 0.000 | 0.000 | 0.000 | 2.162 |
| hexane | 0.000 | 0.000 | 0.000 | 0.000 | 2.668 |
| 3-methylpentane | 0.000 | 0.000 | 0.000 | 0.000 | 2.581 |
| 2,2-dimethylbutane | 0.000 | 0.000 | 0.000 | 0.000 | 2.352 |
| heptane | 0.000 | 0.000 | 0.000 | 0.000 | 3.130 |
| octane | 0.000 | 0.000 | 0.000 | 0.000 | 3.677 |
| 2,2,4-trimethylpentane | 0.000 | 0.000 | 0.000 | 0.000 | 3.106 |
| nonane | 0.000 | 0.000 | 0.000 | 0.000 | 4.182 |
| decane | 0.000 | 0.000 | 0.000 | 0.000 | 4.686 |
| cyclopentane | 0.263 | 0.100 | 0.000 | 0.000 | 2.477 |
| cyclohexane | 0.305 | 0.100 | 0.000 | 0.000 | 2.964 |
| methylcyclohexane | 0.244 | 0.100 | 0.000 | 0.000 | 3.323 |
| cycloheptane | 0.350 | 0.100 | 0.000 | 0.000 | 3.704 |
| cyclooctane | 0.413 | 0.100 | 0.000 | 0.000 | 4.329 |
| pent-1-ene | 0.093 | 0.080 | 0.000 | 0.070 | 2.047 |
| hex-1-ene | 0.078 | 0.080 | 0.000 | 0.070 | 2.572 |
| cyclohexene | 0.395 | 0.200 | 0.000 | 0.070 | 3.021 |
| hept-1ene | 0.092 | 0.080 | 0.000 | 0.070 | 3.063 |
| oct-1-ene | 0.094 | 0.080 | 0.000 | 0.070 | 3.568 |
| dec-1-ene | 0.093 | 0.080 | 0.000 | 0.070 | 4.554 |
| pent-1-yne | 0.172 | 0.230 | 0.120 | 0.120 | 2.010 |
| hex-1-yne | 0.166 | 0.220 | 0.100 | 0.120 | 2.510 |
| hept-1-yne | 0.160 | 0.230 | 0.120 | 0.100 | 3.000 |
| oct-1-yne | 0.155 | 0.220 | 0.090 | 0.100 | 3.521 |
| benzene | 0.610 | 0.520 | 0.000 | 0.140 | 2.786 |
| toluene | 0.601 | 0.520 | 0.000 | 0.140 | 3.325 |
| ethylbenzene | 0.613 | 0.510 | 0.000 | 0.150 | 3.788 |
| *o*-xylene | 0.663 | 0.560 | 0.000 | 0.160 | 3.939 |
| *m*-xylene | 0.623 | 0.520 | 0.000 | 0.160 | 3.839 |
| *p*-xylene | 0.613 | 0.520 | 0.000 | 0.160 | 3.839 |
| styrene | 0.849 | 0.650 | 0.000 | 0.160 | 3.856 |
| *α*-methylstyrene | 0.851 | 0.640 | 0.000 | 0.190 | 4.290 |
| thiophene | 0.687 | 0.560 | 0.000 | 0.150 | 2.943 |
| pyridine | 0.631 | 0.840 | 0.000 | 0.520 | 3.022 |
| methanol | 0.278 | 0.440 | 0.430 | 0.470 | 0.970 |
| ethanol | 0.246 | 0.420 | 0.370 | 0.480 | 1.485 |
| propan-1-ol | 0.236 | 0.420 | 0.370 | 0.480 | 2.031 |
| propan-2-ol | 0.212 | 0.360 | 0.330 | 0.560 | 1.764 |
| butan-1-ol | 0.224 | 0.420 | 0.370 | 0.480 | 2.601 |
| butan-2-ol | 0.217 | 0.360 | 0.330 | 0.560 | 2.338 |
| 2-methyl-propan-1-ol | 0.217 | 0.390 | 0.370 | 0.480 | 2.413 |
| *tert*-butanol | 0.280 | 0.300 | 0.310 | 0.600 | 1.963 |
| pentan-1-ol | 0.219 | 0.42 | 0.370 | 0.480 | 3.106 |
| water | 0.000 | 0.45 | 0.820 | 0.350 | 0.260 |
| methyl acetate | 0.142 | 0.640 | 0.000 | 0.450 | 1.911 |
| methyl propanoate | 0.128 | 0.600 | 0.000 | 0.450 | 2.431 |
| methyl butanoate | 0.106 | 0.600 | 0.000 | 0.450 | 2.943 |
| ethyl acetate | 0.106 | 0.620 | 0.000 | 0.450 | 2.314 |
| vinyl acetate | 0.223 | 0.640 | 0.000 | 0.430 | 2.152 |
| tetrahydrofuran | 0.289 | 0.520 | 0.000 | 0.480 | 2.636 |
| 1,4-dioxane | 0.329 | 0.750 | 0.000 | 0.640 | 2.892 |
| *tert*-butyl methyl ether | 0.024 | 0.210 | 0.000 | 0.590 | 2.372 |
| *tert*-butyl ethyl ether | -0.020 | 0.160 | 0.000 | 0.600 | 2.720 |
| *tert*-amyl methyl ether | 0.050 | 0.210 | 0.000 | 0.600 | 2.916 |
| diethyl ether | 0.041 | 0.250 | 0.000 | 0.450 | 2.015 |
| di-*n*-propyl ether | 0.008 | 0.250 | 0.000 | 0.450 | 2.954 |
| di-*iso*-propyl ether | -0.060 | 0.160 | 0.000 | 0.580 | 2.530 |
| di-*n*-butyl ether | 0.000 | 0.250 | 0.000 | 0.450 | 3.924 |
| acetone | 0.179 | 0.700 | 0.040 | 0.490 | 1.696 |
| pentan-2-one | 0.143 | 0.680 | 0.000 | 0.510 | 2.755 |
| pentan-3-one | 0.154 | 0.660 | 0.000 | 0.510 | 2.811 |
| propanal | 0.196 | 0.650 | 0.000 | 0.450 | 1.815 |
| butanal | 0.187 | 0.650 | 0.000 | 0.450 | 2.270 |
| acetonitrile | 0.237 | 0.900 | 0.070 | 0.320 | 1.739 |
| 1-nitropropane | 0.242 | 0.950 | 0.000 | 0.310 | 2.894 |
